# Reconstructing anatomy from electro-physiological data

**DOI:** 10.1016/j.neuroimage.2017.06.049

**Published:** 2017-12

**Authors:** J.D. López, F. Valencia, G. Flandin, W. Penny, G.R. Barnes

**Affiliations:** a SISTEMIC, Engineering Faculty, Universidad de Antioquia UDEA, Calle 70 No. 52-21, Medellín, Colombia; b Solar Energy Research Center SERC-Chile, Department of Electrical Engineering, University of Chile, Santiago, Chile; c Wellcome Trust Centre for Human Neuroimaging, Institute of Neurology, UCL, 12 Queen Square, WC1N 3BG, London, UK

**Keywords:** MEG/EEG brain imaging, Brain anatomy, Negative variational free energy, Fourier spherical harmonics

## Abstract

Here we show how it is possible to make estimates of brain structure based on MEG data. We do this by reconstructing functional estimates onto distorted cortical manifolds parameterised in terms of their spherical harmonics. We demonstrate that both empirical and simulated MEG data give rise to consistent and plausible anatomical estimates. Importantly, the estimation of structure from MEG data can be quantified in terms of millimetres from the true brain structure. We show, for simulated data, that the functional assumptions which are closer to the functional ground-truth give rise to anatomical estimates that are closer to the true anatomy.

## Introduction

Imaging brain structure and electrophysiology is typically a two-stage process. Structure is estimated from a magnetic resonance imaging (MRI) scan whereas function is derived from magneto- or electro-encephalographic (MEG/EEG) data. MEG/EEG data are desirable as they are passive (no energy passes into the subject) and non-invasive measurements of neuronal current flow with high temporal resolution (millisecond). The estimation of cortical current flow underlying the MEG/EEG signal is however an ill-posed problem because the solutions are non-unique –meaning that many different current distributions could explain the same MEG/EEG data equally well. For the remainder of this paper we restrict our discussion to the MEG case.

**Fig. 1:**
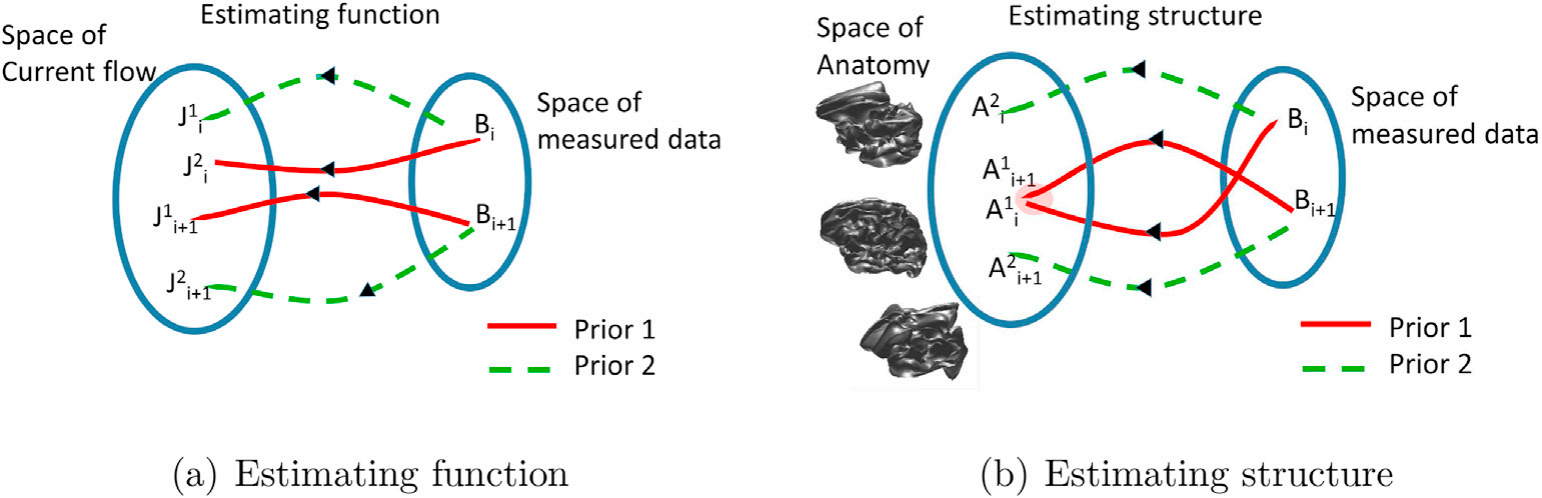
(a) Shows a one-many mapping for different task data. For any task (or period of time) *i* the same MEG data (*B*) can be described by more than one current distribution (*J*). The current distribution that is estimated depends on prior functional assumptions (1 or 2, which could correspond to minimum norm and beamformer for example). At a different time (or task) i+1, new MEG data can be explained by another quite distinct set of possible current distributions. (b) Shows the many-one mapping one should expect from estimating anatomy. In this case although there will be many different possible anatomical structures which could underlie any one set of measurements $B_{i}$, this set of structures must be close to the set explaining the data at time $B_{i+1}$. Indeed, as more MEG data is recorded, the space of anatomy that could explain all of the data gets smaller and smaller. As the anatomy is contingent on reasonable functional priors, only the correct functional assumptions (and forward models) will lead us to the true anatomy.

One common way to reduce the set of allowable solutions is to constrain current flow to lie on the subject's cortical grey matter surface derived from MRI (Dale and Sereno, 1993). This model is used to estimate the location and time-series of the sources of neural activity by solving an inverse problem (Fig. 1(a)). As the problem is ill-posed, additional functional assumptions (or priors) are necessary. These functional assumptions generally take the form of a minimum energy constraint in addition to a constraint on the underlying source covariance structure (Mosher et al., 2003, Friston et al., 2008b, Wipf and Nagarajan, 2009). For example, the minimum norm algorithm (Hämäläinen and Ilmoniemi, 1994) is characterized by a diagonal source covariance matrix; LORETA (Pascual-Marqui et al., 1994) has a source covariance matrix with a broader diagonal structure reflecting an intrinsically smooth distribution of the cortical surface, and certain algorithms –such as Multiple sparse priors (MSP), create this covariance structure through the superposition of sparse, but locally smooth, patches of cortex (Friston et al., 2007) (see Baillet et al., 2001, Wipf and Nagarajan, 2009 and Henson et al. (2011) for overviews of the field).

Here we turn this approach around and ask which is the most likely cortical surface given the MEG data and a specific set of functional priors. We do this by searching over a space of possible cortical surfaces parameterised in terms of spherical harmonics: higher harmonics describing finer spatial structure (Chung et al., 2007). In previous work (Stevenson et al., 2014), we showed that the ability to discriminate distorted from true anatomy (by removing higher harmonics) could be used as a metric of spatial accuracy for source reconstruction. In this work we use the harmonic structure to define a space of brain shapes, each containing the same amount of spatial detail, and use the MEG data select the most appropriate anatomy. We take advantage of the considerable natural co-variation of these harmonics by using a library of cortical surfaces, and decomposing the ensuing basis functions into an orthogonal set of features using Singular Value Decomposition (SVD). This space is defined based on a library of normal brains. Within each harmonic order, we can define a canonical vector that describes the direction of most of the variability over the library of brains. In this work we take two such canonical vectors from two different harmonic orders in order to create a two-dimensional space of brain surfaces (this coordinate frame is arbitrary -see discussion). This considerably reduces (or effectively regularizes) the complexity of the problem to fewer features than brains in the dictionary.

There are two main themes to this paper. The first is to show that it is possible to reconstruct the shape of an individual's cortical surface based on MEG data. The second, is to show how such anatomical estimates can be used to refine the search for optimal functional priors. Specifically, in MEG we have very little ground truth (whole-brain electrophysiological recordings) to which to compare our functional estimates, in addition every new task gives rise to a different functional estimate (Fig. 1(a)). In contrast however, all functional task data derive from the same anatomy –we predict that ideal set of functional assumptions would deliver anatomical estimates that continue to improve as more tasks are studied (Fig. 1(b)).

The paper proceeds as follows. We introduce the space of anatomy and the Bayesian formalism behind the approach. We demonstrate how, given an appropriate basis set of cortical anatomies, it is possible to use MEG data to estimate individual cortical structure. We show how this estimate is robust across recording runs. We show how the Free energy or model evidence for each candidate surface supporting the MEG data covaries with the distance of that candidate surface to the subject's true anatomy (unknown to the algorithm). We show that such anatomical estimates are robust for real and simulated data. Importantly, we also show that the estimation of the correct (or closest anatomy to that extracted from a structural MRI scan) anatomy is contingent on the choice of appropriate functional prior.

## Methods

The guiding assumption (backed up by previous studies: (López et al., 2012, Stevenson et al., 2014, Martínez-Vargas et al., 2016, Troebinger et al., 2015, Meyer et al., 2017)) is that a generative model of the MEG data based on a cortical surface different from the one generating the data will typically have a higher model complexity as measured by the model evidence. We have access to this relative decrease in the model evidence as approximated by negative variational Free energy (Friston et al., 2007, López et al., 2014). We first detail how the space of cortical surfaces is formed based on anatomy from many subjects, and then go on to describe how it is then possible to score anatomical models, within this space, for any single subject MEG data.

### Fourier spherical harmonic representation of the cortical surface

The cortical surface can be decomposed into an orthogonal basis set of spherical harmonic components expressed as a weighted linear combination of Fourier coefficients. Pial surface meshes can be extracted from a structural MRI (sMRI) using FreeSurfer (Fischl, 2012) software package, and a weighted Fourier series (WFS) representation can be computed as in Chung et al. (2008). This weighted Fourier representation begins with an ellipsoid and progressively builds up spatial detail as the number of spherical harmonics (degree) is increased. For a surface of degree *c* there are a total of ${\left(c+1\right)}^{2}$ Fourier coefficients to estimate for each of the axes per hemisphere (Chung et al., 2008).

A set of up to $N_{b}=27$ brains acquired with sMRI were used to generate the library of candidate brains. Each cortical surface was down-sampled to a set of $N_{d}=21,401$ vertices. The WFS representation was generated with a degree of c=20 which, based on past experience (López et al., 2013, Stevenson et al., 2014), is the point at (or just beyond) which the Free energy of current estimates on these surfaces begins to saturate. This gave a vector of Fourier components per harmonic order per subject describing the cortical surface at a specific spatial scale.

In order to demonstrate proof-of-principle we choose to modulate brain shape in two dimensions by perturbing two arbitrary (see discussion) harmonic orders (the $6^{\text{th}}$ and $9^{\text{th}}$). We did this by taking the first eigenmode of variation over the Fourier coefficients within this order (mean corrected), and then stepping along this vector by multiples of the standard deviation over subjects.

### Model reduction with SVD

The estimation of the 2646 coefficients ($6{\left(c+1\right)}^{2}$ with c=20) describing the fully parameterised surface from MEG data alone would be challenging. Here we make two simplifications: (*i*) we select just two spherical harmonic degrees as dimensions along which to optimize, and (*ii*) within each degree we work only along the principal eigenmode of parameter variation.

For each harmonic order *j* there will be (2j−1) Fourier coefficients for each of 3 dimensions (x,y,z) over two hemispheres, i.e., in total $N_{w}=6\left(2j-1\right)$ coefficients per subject. These can be concatenated over $N_{b}$ subjects into a matrix $G_{j}\in {ℜ}^{N_{b}\times N_{w}}$. So that for example, $G_{1}$ contains a list of the mean locations of each subject's hemisphere, $G_{2}$ describes the best fitting ellipsoids to these hemispheres, and as *j* increases $G_{j}$ corresponds to the coefficients adding increasingly fine spatial detail.

For each order *j*, we first remove the mean from each row (or subject):

(1) $$\hat{G}=G-\bar{G}$$

with $\bar{G}\in {ℜ}^{1\times N_{w}}$ a vector with the mean of each coefficient over subjects. We then extract the first eigenmode describing the variation of coefficients (within this harmonic degree) over subjects: ${\hat{G}}^{T}\hat{G}=USV^{T}$, with ${\left(\cdot \right)}^{T}$ the transpose operator. Now move the new coefficients along the dominant eigenmode $U_{\cdot ,1}$, with sub-index (⋅,1) indicating the first column of matrix *U*; in steps of size *δ* scaled by the eigenvalue magnitude $\sqrt{S_{1,1}}$, where $s=\delta \sqrt{S_{1,1}}$ is a fractional step of the dominant eigenvalue. Here we use δ={−2,−1,0,1,2} as a coarse scale, and δ={−2,−1.5,…,2} as a fine one.

This gives a displaced set of Fourier coefficients for this harmonic order $X\in {ℜ}^{1\times N_{w}}$:

(2) $$X=SU_{\left(\cdot ,1\right)}+\bar{G}$$

As all brains were created by expanding the same single unit sphere (using different sets of Fourier coefficients) we were able to quantify the physical distance between any two brains as the mean Euclidean distance between vertex pairs (where each vertex shares the same index on the two different meshes). This method is symmetric and unbiased but does result in a relatively small range of values describing large variation in brain shape (see Supplementary Figs. S1 and S2).

### Free energy for model selection

The use of spherical harmonics allows us to create a search space of cortical structure (each with a different lead-field matrix) formed with a combination of Fourier coefficients. The next step is to quantify which of these structures most likely underlies the measured MEG data.

For a MEG dataset $Y\in {ℜ}^{N_{c}\times N_{t}}$ of $N_{c}$ sensors and $N_{t}$ time samples, the magnitude of the neural activity $J\in {ℜ}^{N_{d}\times N_{t}}$ on a mesh of $N_{d}$ current dipoles distributed over the cortical surface can be represented by the general linear model (Dale and Sereno, 1993):

(3) $$Y=L_{a}J+\varepsilon$$

with additive noise *ε*. Solving the M/EEG inverse problem gives an estimate $\hat{J}$ that is strongly dependent on the assumptions about anatomy *a*, embodied in the gain (or lead-field) matrix $L_{a}$ (where current is assumed to flow normal to the cortical surface). The (negative variational) Free energy (Friston et al., 2007) approximates the log of the model evidence, F≈log(p(Y)) and is a trade-off between the model accuracy and complexity (Penny, 2012).

Following the methods outlined in López et al. (2012), the posterior over source space estimates via Bayes’ rule may eliminate the dependency on the anatomical model just by performing two steps:

1. Current source density *J* is estimated deterministically to produce the posterior over neural activity given data and anatomical assumptions:

(4) $$p\left(J|Y,a\right)=\frac{p\left(Y|J,a\right)p\left(J|a\right)}{p\left(Y|a\right)}$$

Without prior information both the likelihood and the prior over the neural activity are proposed as multivariate normal density functions $p\left(Y|J,a\right)=N\left(Y;L_{a}J,Q_{\varepsilon }\right)$ and $p\left(J|a\right)=N\left(J;0,Q_{a}\right)$; with $Q_{\varepsilon }$ and $Q_{a}$ the sensor noise and source space covariances, respectively.

2. The likelihood of anatomical parameters p(Y|a) gives the model dependent evidence in Eq. (4). The posterior over parameters can be computed by pooling over all anatomical models available:

(5) $$p\left(a|Y\right)=\frac{p\left(Y|a\right)p\left(a\right)}{\sum_{r}p\left(Y|a_{r}\right)p\left(a_{r}\right)}$$

for $r=1,\ldots ,N_{a}$ models, where the prior of each model $p\left(a_{r}\right)$ is given by the variability of the coefficients within the set. In reality, this prior would be higher for brain shapes that were more likely to occur. In this work however, in order to demonstrate that the MEG data alone are selective of the true cortical structure, the prior is uniform over all models in the space.

For each new surface, a new lead-field matrix $L_{a}$ was computed with the SPM12 software package (http://www.fil.ion.ucl.ac.uk/spm/), using the Nolte Single Shell forward model (Nolte, 2003).

In this paper, we make use of the Empirical Bayes beamforming (EBB) priors (Belardinelli et al., 2012). Briefly, there is a single empirical prior covariance matrix estimated directly from the data under beamforming assumptions. Then, solving Eq. (4) the estimated current density ${\hat{J}}_{a}$ for each anatomical model is computed with:

(6) $${\hat{J}}_{a}=Q_{a}L_{a}^{T}{\left(L_{a}Q_{a}L_{a}^{T}+Q_{\varepsilon }\right)}^{-1}Y$$

where $Q_{\varepsilon }$ is the sensor level noise covariance. The optimal values of ${\hat{J}}_{a}$ are obtained with an expectation-maximisation algorithm using Free energy as the objective function (Friston et al., 2008b). This means that each estimated current density ${\hat{J}}_{a}$ has an associated Free energy value $F_{a}$ (see López et al. (2014) for more implementation details), which is computed as (Friston et al., 2007):

(7) $$F_{a}=-\frac{N_{t}}{2}\text{tr}\left({\text{Σ}}_{Y}{\text{Σ}}_{a}^{-1}\right)-\frac{N_{t}}{2}\text{log}|{\text{Σ}}_{a}|-\frac{N_{t}N_{c}}{2}\text{log}\left(2\pi \right)-\frac{1}{2}{\left(\hat{\lambda }-\upsilon \right)}^{T}\text{Π}\left(\hat{\lambda }-\upsilon \right)+\frac{1}{2}\text{log}|{\text{Σ}}_{\lambda }\text{Π}|$$

where |⋅| is the matrix determinant operator, ${\text{Σ}}_{Y}=\frac{1}{N_{t}}YY^{T}$ is the data covariance, and ${\text{Σ}}_{a}=Q_{e}+L_{a}Q_{a}L_{a}$ is the model based covariance at sensor level (after the expectation-maximisation optimisation). For each estimated current density ${\hat{J}}_{a}$, the final Free energy value $F_{a}$ is obtained after optimising the hyperparameters *λ* that provide a trade-off between the sensor noise ($Q_{\varepsilon }=\lambda_{1}I_{N_{c}}$) and the Beamforming prior $Q_{a}=\lambda_{2}{\left(L_{a}^{T}{\left(YY^{T}\right)}^{-1}L_{a}\right)}^{-1}$. The prior and posterior distributions of the hyperparameters are considered Gaussian: $q\left(\lambda \right)=N\left(\lambda ;\upsilon ,{\text{Π}}^{-1}\right)$ and $p\left(\lambda \right)=N\left(\lambda ;\hat{\lambda },{\text{Σ}}_{\lambda }\right)$ respectively (Friston et al., 2008b). For the specific case of Beamformers, the relation between $\lambda_{1}$ and $\lambda_{2}$ is also known as the regularisation parameter (Golub et al., 1979).

It is expected that the model *a* corresponding to the solution with maximum Free energy will be the most probable López et al. (2012).

The code for the simulations and inversions is available here.^1^

## Materials

The task we selected for validation consisted of abductions of the right hand index finger to an auditory cue as originally described in Muthukumaraswamy (2011) and presented in Troebinger et al. (2014). We recorded 8 runs of ten minutes (of approximately 145 trials each) from a single male subject wearing a head-cast, spread over three sessions on separate days. Briefly, MEG data were collected using a 275 channel CTF Omega system at 600 Hz sampling rate with 150 Hz anti-aliasing filters. The cue consisted of a simple auditory tone (1 000 Hz), played via a piezo electric device connected via plastic tubing to ear-inserts, followed by an inter-stimulus interval of 3.4–4.5 s. Thresholded rectified EMG traces of the first dorsal interosseus (FDI) were used to generate a trigger locked to movement onset (time 0 in the plots). The trials were baseline corrected to the 50 ms pre-movement onset period. The source estimates were made using the EBB algorithm (Belardinelli et al., 2012) over a [0–48] Hz bandwidth and [-300 to +400] ms window around the trigger based on averaged data (one average for each of the eight recording runs). The cortical surface of subject who performed the task was not part of the original library of brains.

## Results

**Fig. 2:**
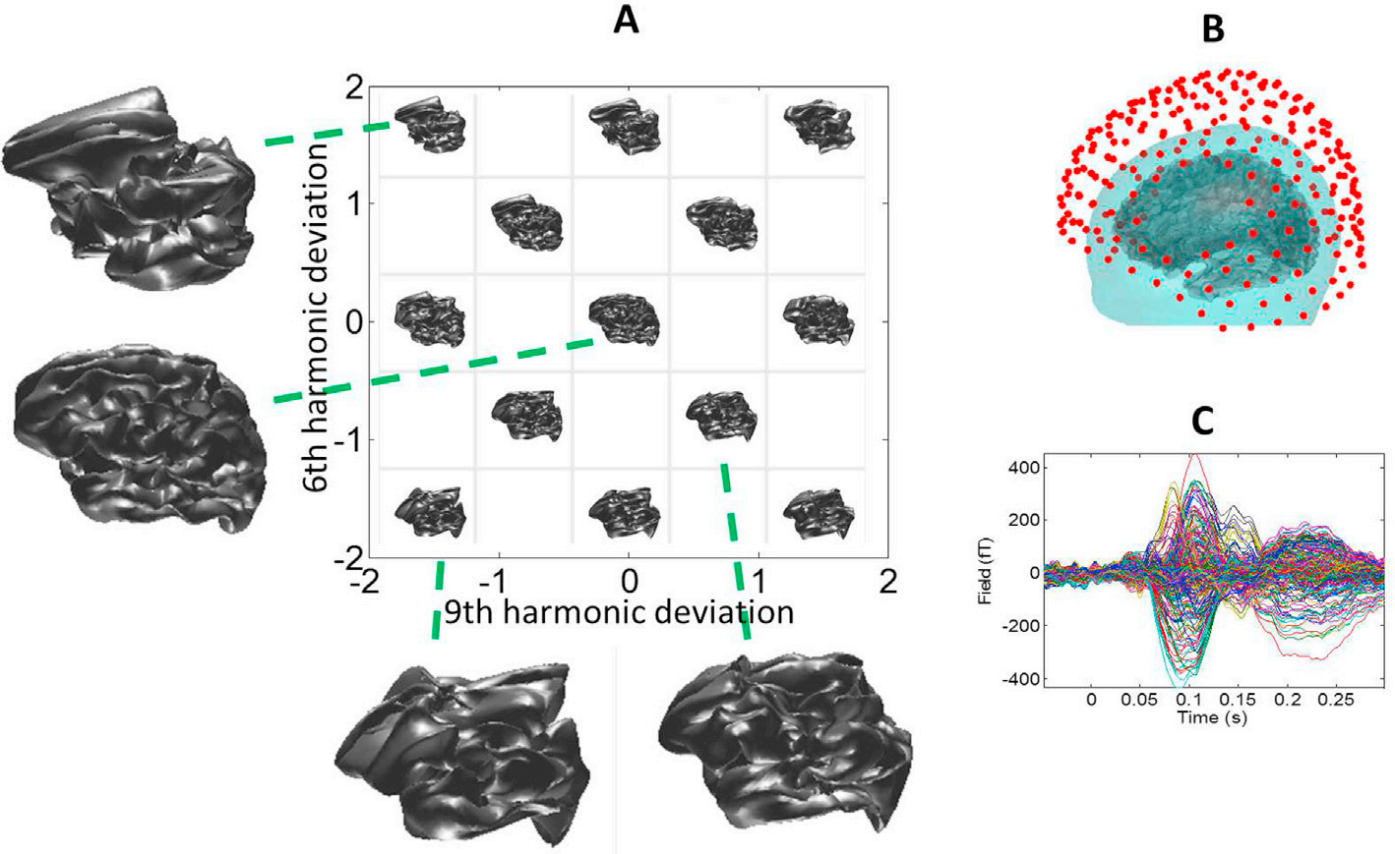
A) Two dimensional space of candidate brains corresponding to distortions in the 6th and 9th spherical harmonics by two standard deviations of normal variation. The surface at the origin (central image) is the average brain (over subjects in anatomical database), and caricature brains develop for increasing distortions. B) Schematic of the MEG sensors measuring magnetic field change around the subject's head. C) Average change (over trials, from 274 sensors) in magnetic field due to current flow in the subject's cortex (taken to be an unknown structure) time-locked to a finger movement.

In Fig. 2(A) we move along two canonical vectors (within the 6th and 9th spherical harmonic components) from a 20 dimensional cortical space. The cortical surfaces move from average (0,0) to caricature brains at the edges (two standard deviations from the mean) of the space. Fig. 2(C) shows the MEG data due to a cued finger movement of a subject whose brain is not in the anatomical database. We now estimate the cortical current flow due to this finger movement on each of the possible cortical surfaces (Fig. 2(A)), and compute the Bayesian model evidence for each reconstruction. The logic is that when the underlying anatomy is incorrect, a more complicated (and therefore less likely) current distribution is expected to be required to explain the same data.

**Fig. 3:**
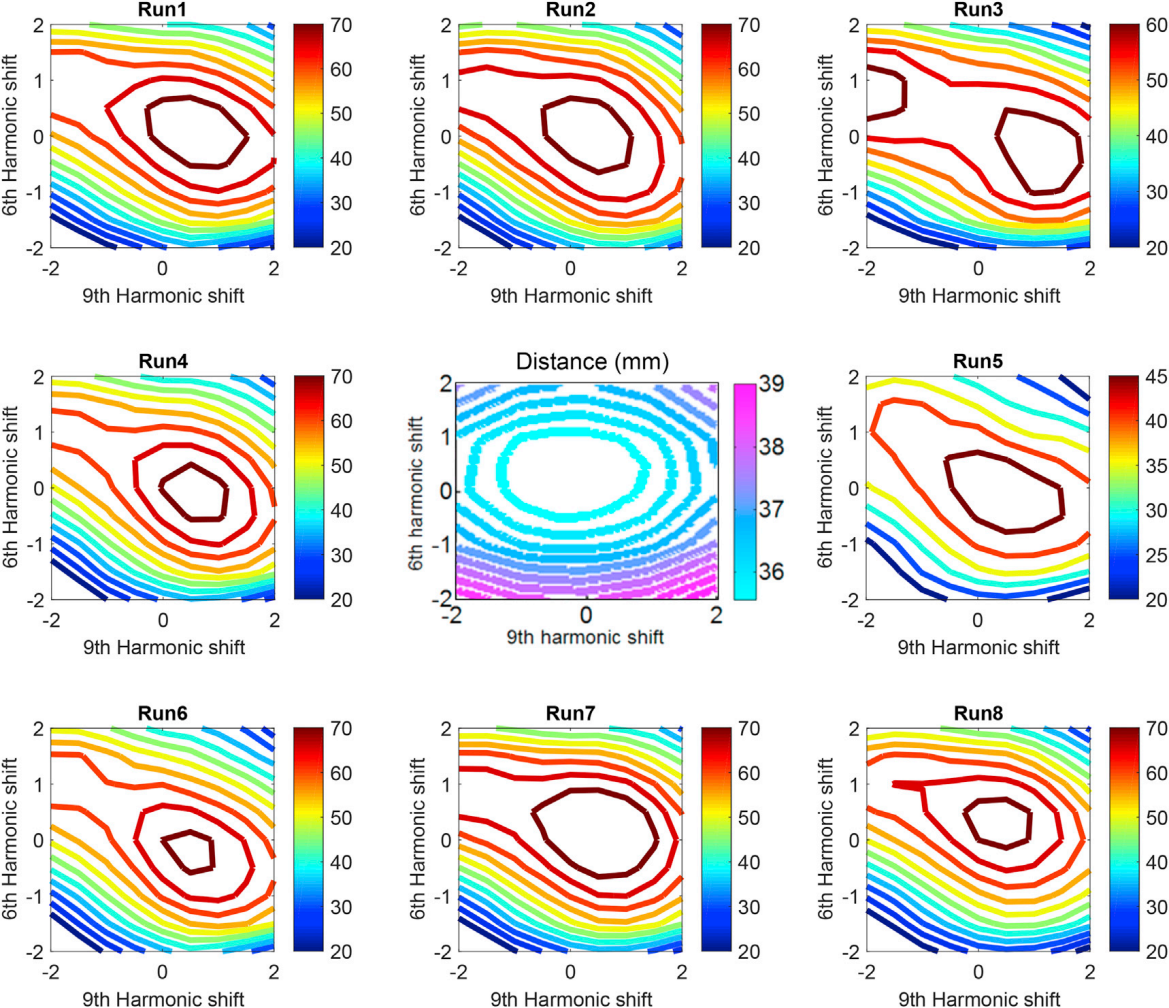
Contour plots around the edges of the figure show the Free energy obtained by solving the MEG inverse problem with eight different datasets over a grid of 81 distorted candidate brains. All datasets (except the top right panel) give rise to a similar global maximum close to the origin (or average brain shape) showing the stability of the approach to initial conditions. The centre panel shows the independently computed mean RMS error (in mm) between the space of deformed brains and the true brain structure (which is being estimated in the surrounding panels).

Fig. 3 shows Free energy across the space of candidate brains for eight datasets of the finger-abduction task. All Free energy values were obtained based on beamforming priors and a dataset over a grid of 81 distorted brains (shifting harmonics 6 and 9). All results except one (upper right in Fig. 3) had a single convex global maximum close to the origin (or average brain shape). The middle plot shows the independently calculated Root Mean Square Euclidean distance between (every vertex in) each brain in the harmonic space and the corresponding vertex on the participant's cortical surface (unknown to the algorithm). Note that the algorithm had no knowledge of true brain structure and corresponding distance metric (central plot) yet the most likely cortical surfaces (maxima in Free energy plots) are those close to the true brain (the distance metric minimum). It is also noteworthy that the anatomical solution (the peak in the Free energy function or the most likely candidate brain) is robust across recording runs, i.e., robust to initial conditions.

**Fig. 4:**
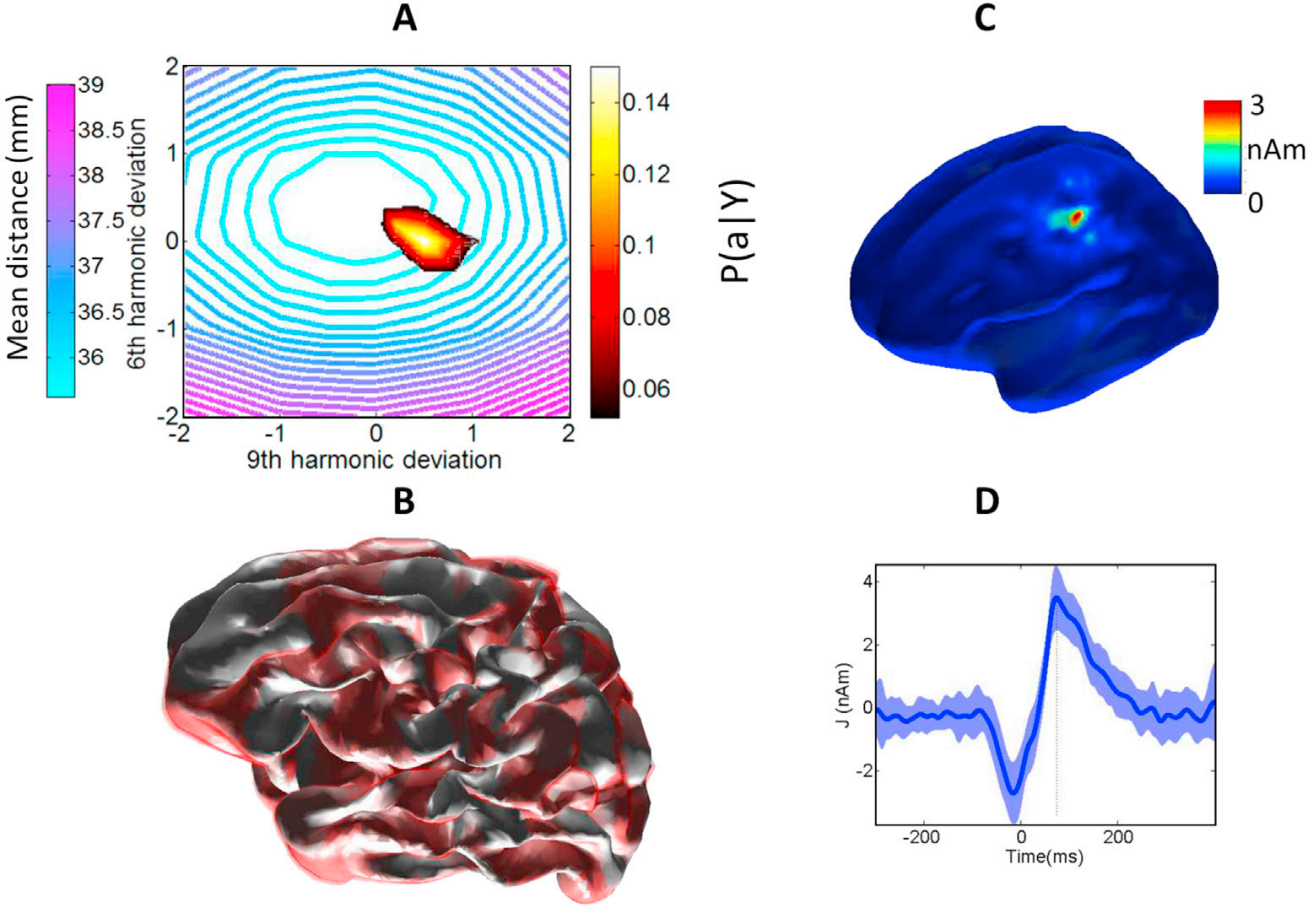
A) Hot colours (red/white) show the 95% posterior probability estimate of cortical structure based on the MEG data; cool colours (blue/purple) show the average Euclidean distance from points in the true brain structure to corresponding points in each brain in the library. Panel B) shows the most likely anatomical model given the MEG data (the cortical structure at the peak of the probability distribution in panel A); the red contour shows the 95% confidence interval for this structural estimate. C) Shows estimated electrical activity on the inflated cortical surface (from panel B) at t=72 ms post button press. D) Shows the time-course of this activity (extracted from MNI location -46, -16, 50 mm); the shaded region shows the 95% confidence intervals on this estimate.

Fig. 4(A) shows a condensed version of the above with the probability of each surface given the MEG data as shown by the density map (hot colours). Note that the most likely brain structures based on the functional data (hot colours) correspond with those brain structures closest to the true underlying anatomy (cold colours). Ideally, the functional estimate of brain structure would peak at the same point as the minimum physical distance between brain structures. It is important to note that this subject's brain was not in the original database (and their brain is not the ideal average brain); therefore, there is no point in this 2D space which perfectly reconstructs the cortex (hence the distance metric never reaches zero). In addition to this we are using just 2 dimensions to approximate a solution to a much higher dimensional optimization problem. The range of distances are large and compressed within a narrow range (35–39 mm) as we were aiming for an unbiased and symmetric distance metric. The metric is based on comparing distances between vertices with the same indices on the initial unit sphere (which was deformed to make all surfaces). This means that the distance metric is conservative and very sensitive to cortical folding patterns (see Supplementary Fig. 1 and Discussion). Supplementary Fig. 2 shows the functional estimate of anatomy alongside the nearest-neighbour and Hausdorf distance metrics.

Fig. 4B shows the most likely cortical structure given the MEG data along with red-bands showing the confidence interval on this anatomical estimate. Note that we have made no a-priori bias towards reasonable brain structures here. Importantly, as the reconstruction of anatomy is contingent on sensible functional assumptions, we now have some confidence in the current flow estimation shown over space (of the estimated cortical structure) in Fig. 4(C) and over time in Fig. 4(D).

### Control conditions

**Fig. 5:**
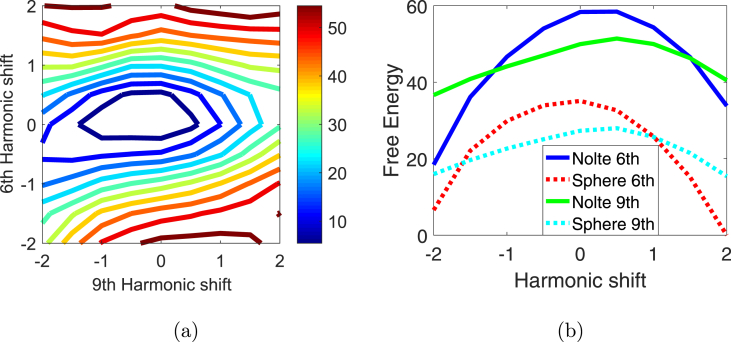
Control conditions. (a) Estimate of structure (point of maximal Free energy) using very low SNR data (the data from Fig. 3, run 1, filtered between 100 and 200 Hz). In this case the plausible brain structures –close to (0,0)– are the least probable. (b) Shows the two different contour plot dimensions in 1D for the sensitivity to anatomy using either the Nolte single shell model (as above), or a single sphere model. Note that both forward models peak at the same approximate anatomical location (but that the Nolte model is much more likely).

To confirm that there was no intrinsic bias in our method towards the true cortical surface, we applied the same algorithm to the same MEG data but filtered into a band with very little physiological signal (from 100 to 200 Hz). Fig. 5(a) shows, for these noisy data, that structures closest to the true brain (the minimum of the distance metric in Fig. 4) in this two dimensional space become the least likely, demonstrating that there is no intrinsic bias in our method towards plausible looking brains. A related confound might be that the Nolte Single shell model used here makes use of the inner skull boundary to construct the forward model -one could argue that the method is therefore biased towards surfaces which fit within this boundary. For this reason we replaced the Nolte model with a single sphere model (where the only anatomical information used by the forward model is the centre of the head-approximating sphere). Note that we get very similar dependence on anatomy for both forward models (Fig. 5(b)) –ruling out the possibility that there are structural clues in the forward model -but also that the Nolte model provides a much more likely explanation showing data.

**Fig. 6:**
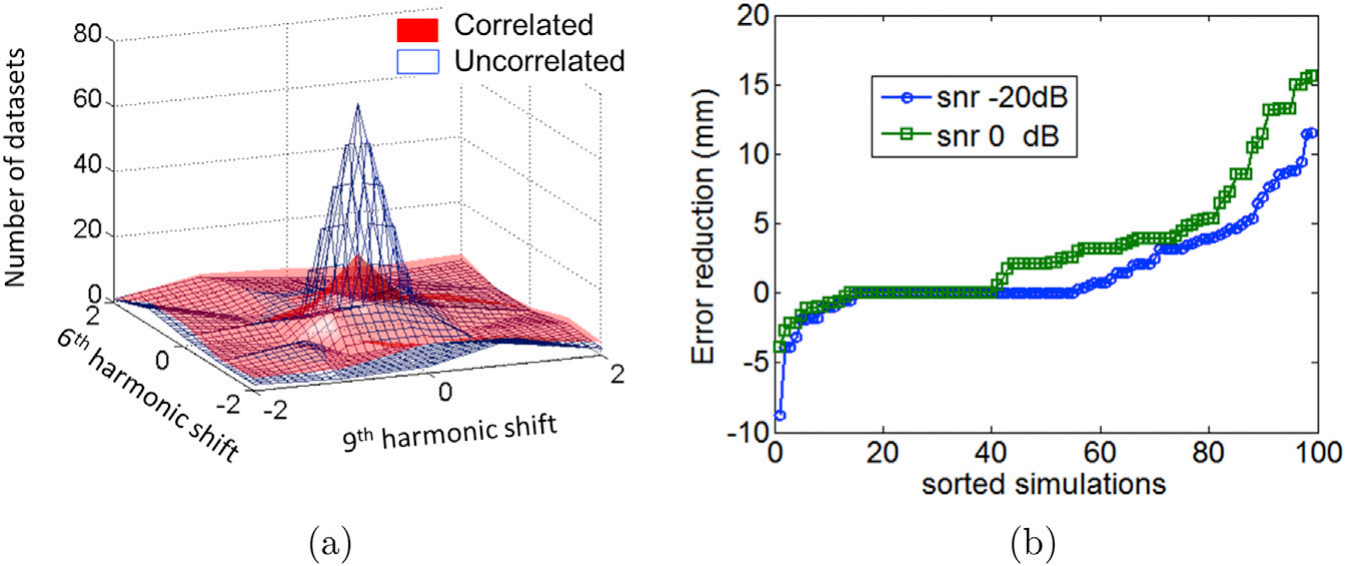
Anatomical reconstructions of 200 datasets (100 correlated and 100 uncorrelated source pairs) simulated on the cortical surface at the origin of the space of brains (in Fig. 2). (a) Shows the histogram of the most likely anatomy when data were reconstructed using beamformer (uncorrelated) priors for correlated (red) and uncorrelated (open mesh) source pairs at 0 dB SNR. (b) Shows the (sorted) reduction in anatomical reconstruction error (in mm) when the anatomy was reconstructed based on uncorrelated rather than correlated source pairs (at same location and SNR) for -20 (blue squares) and 0 dB (green circles) SNR. For example, at 0 dB SNR anatomical reconstruction accuracy improved in all but 15% of cases when the source pairs were uncorrelated (congruent with the functional prior). This contrast (between appropriate and inappropriate priors) in anatomical reconstruction accuracy was less marked at lower SNR.

Finally we wanted to test our conjecture that functional priors that are closer to reality will give rise to converging and more accurate structural estimates. In order to do this we simulated multiple datasets (100 per condition and SNR) each containing a pair of sources that were either temporally correlated or uncorrelated at two different SNRs -20 and 0 dB. The source locations and SNRs were identical across correlated and uncorrelated conditions and all source simulated on the brain structure at the origin of our space (Fig. 2). We then made an estimate of the anatomy from each of these datasets using an EBB (Empirical Bayes Beamformer) algorithm (Belardinelli et al., 2012). The functional prior here is that cortical sources are uncorrelated. Fig. 6 (a) shows the histogram of estimated anatomy for the two different kinds of data –for data which does not match the beamforming assumptions (correlated, red surface) there is no strong preference for any anatomy, whereas for data which are congruent with the functional priors (uncorrelated, open mesh) there is a clear preference for the true anatomy (at the origin). For any pair of sources, one can plot how much closer the anatomical estimate gets to the truth when these sources are uncorrelated rather than correlated (Fig. 6(b)). Note that as the SNR increases (0 dB, green circles as compared to −20 dB blue squares) there is more to be gained (i.e. larger reduction in the error to true anatomy) from using the congruent functional priors.

## Discussion

We have demonstrated that information about cortical anatomy can be obtained from empirical electrophysiological data. Framing the MEG inverse problem in the context of anatomy give us an unambiguous metric (millimetres from an MRI derived surface) through which to directly quantify the quality of any MEG source reconstruction. Technologically the approach is interesting as it makes the first step towards a generation of electrophysiological scanners able to deliver both structural and functional information.

We were also able to show, in simulation, that sub-optimal functional priors will give less accurate and more variable anatomical estimates (see Fig. 6). Our conjecture, which remains to be proven or empirically demonstrated, is that, as the repertoire of task data on any one subject is increased, then the best functional assumptions will be those that lead to the smallest (and most accurate) space of anatomy (Fig. 1).

It is important to note that there will be portions of cortex which produce no MEG measurable field. Here we take advantage of the fact that the cortex is a continuous and smoothly varying manifold; and assume that the less visible sections of cortex (for example the crests of gyri) can be interpolated from neighbouring and MEG visible (for example the walls of the sulci) cortex. It also speaks to the inclusion of other modalities (like) EEG to minimize the null-space (see a related approach by Hansen et al. (2016)). It is also clear that MEG data which arise from sources that have not been explicitly modelled (external noise or deep brain structures which are not represented) will add noise to this process.

This work builds on the methods laid down by Henson et al. (2009) who were the first to use model evidence to compare between different forward models. By demonstrating that higher model evidence (Friston et al., 2008a, Penny, 2012, Wipf and Nagarajan, 2009) is associated with better anatomical estimates; here we have again shown this cost function to be extremely powerful. Critically, the monotonic relationship linking Free energy to physical distance error expanded upon here (Fig. 3) and elsewhere (Stevenson et al., 2014) removes the possibility that the higher model evidence (Free energy) values reflect better fitting of un-modelled noise (such as heartbeat or eye-blink artefacts).

We expected the empirical noise data (Fig. 5(a)) to give rise to a flat Free energy surface (much like those observed in simulations –Fig. 6(a)), but the anatomical estimate is very significantly biased away from the true anatomy. We do not fully understand this but it could be a combination of a spatially structured distribution of this noise alongside an over-estimation (by the Free energy estimation) of the amount of signal relative to noise in the data.

There are many ways that this work could be extended, specifically we have used a spherical harmonic approach to define the cortical surface but other parameterisations may well be better suited to this task (Cootes and Taylor, 2004, Wang et al., 2012). Note that if the problem is to estimate anatomy, there is a lot more information at our disposal. Here we made the arbitrary choice of 6th and 9th harmonics. We selected these for a tangible demonstration so as to give a range of structures which clearly looked like brains closer to the origin but were nonsensical away from the origin. Ideally one would optimize over all harmonics, and the finer the spatial detail (the higher the harmonic) the more difficult to reconstruct it accurately (see Stevenson et al. (2014)). Although here we only looked at two harmonics (and hence considerably simplified this high-dimensional optimization) we made very little use of any prior information about brain structure or its normal variation (i.e., our priors over the space of brains were flat). One could make higher dimensional optimization scheme tractable by making use of prior distributions of these features (p(a) in Eq. (5)) based on normal variation.

The distance metric we use here is topologically defined -the brains being compared share the same original spheres which are then distorted to an individual brain shape. We then compute how far an individual vertex in one brain has moved from its partner to another. This means that the metric is very sensitive to different folding patterns between brains. Supplementary Fig. 1 shows the distribution of distances over the cortical surface -the distances being smallest for consistent features like the central sulcus, and largest where folding patterns differ between individuals. Supplementary Fig. 2 shows alternative distance metrics -which might be more appropriate for different applications. The optimization of structure is based on the estimate of a current distribution, so there will be no anatomical information available for regions of cortex not specifically involved in the task (López et al., 2013). Here we are constrained to a motor task, in future one might consider data partitioning strategies (Woolrich et al., 2013, Martínez-Vargas et al., 2016) in order to create estimates of whole-brain anatomy based on resting state data.

## Footnotes

1 https://drive.google.com/a/udea.edu.co/file/d/0B_L4WKmGKjF9eTQzX25XRm9UTkE/view?usp=sharing.
