# Supplementary material for: Reconstructing anatomy from electro-physiological data
Source: Neuroimage. 2017 Dec;163:480–6. doi: 10.1016/j.neuroimage.2017.06.049 (PMC5725312; doi:10.1016/j.neuroimage.2017.06.049)
Supplement: Supplementary file 1 [file mmc1.pdf]

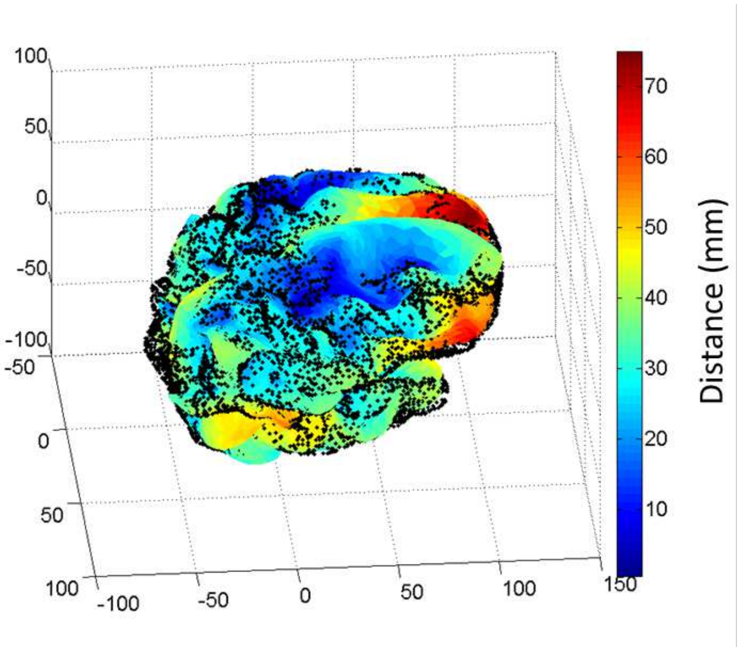

(a) Estimating function

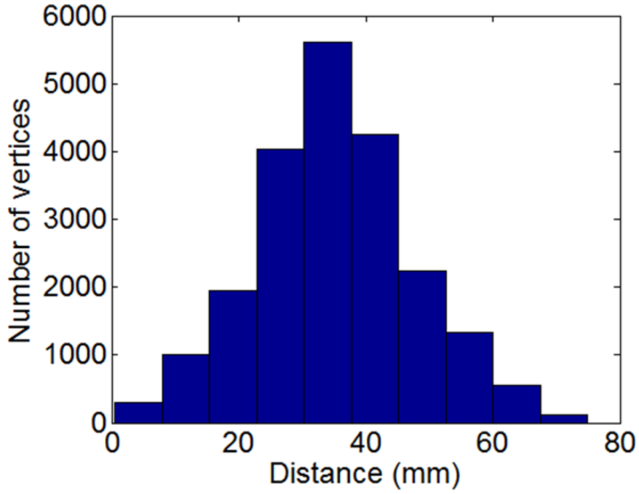

(b) Estimating structure

Figure 1: The corresponding vertex-vertex distances from the subject's brain to the nearest cortical surface from the library. Top panel shows subject's brain in black dots overlaid on distance colour-coded library-brain surface. Lower panel shows distribution of the distances over vertices. The average distance between these two surfaces is 35.39 mm.

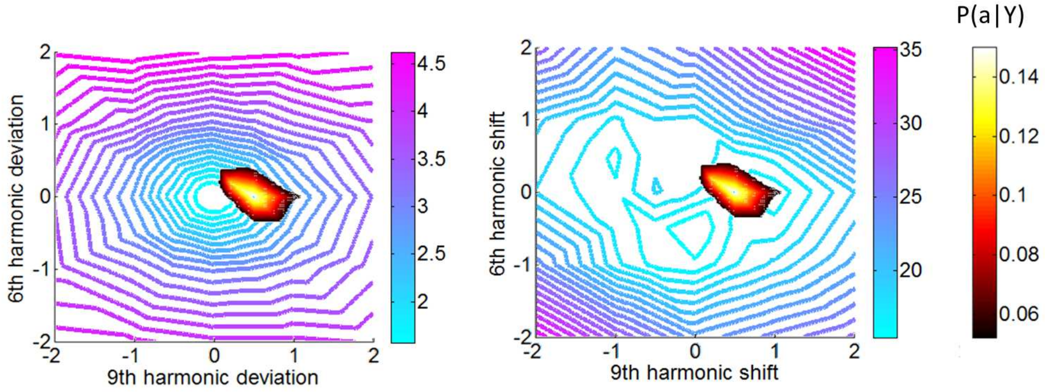

Figure 2: Alternative distance metrics between true brain and space of brain surfaces (cool colours) alongside the functional estimate ($p(a|Y)$, hot colours). The functional estimate is the same as that shown in Fig. 4. Top panel shows the mean nearest-neighbour distance between the subject's brain and the space of brain surfaces. Lower panel shows the Hausdorf distance between the subject's brain and the space of brain surfaces.
